# Association of PTPN22 SNP1858 (rs2476601) and Gene SNP1123 (rs2488457) Polymorphism With Primary Immune Thrombocytopenia Susceptibility: A Meta-Analysis of Case-Control Studies and Trial Sequential Analysis

**DOI:** 10.3389/fgene.2022.893669

**Published:** 2022-05-25

**Authors:** Haokun Tian, Weikai Xu, Lequan Wen, Lirui Tang, Xinyuan Zhang, Tiangang Song, Changsen Yang, Peng Huang

**Affiliations:** ^1^ Joint Program of Nanchang University and Queen Mary University of London, Nanchang, China; ^2^ Center for Evidence-based Medicine, School of Public Health, Nanchang University, Nanchang, China; ^3^ Jiangxi Province Key Laboratory of Preventive Medicine, School of Public Health, Nanchang University, Nanchang, China

**Keywords:** ITP, primary immune thrombocytopenia, gene polymorphism, PTPN22, meta-analysis

## Abstract

**Objective:** Systematic review of the association of protein tyrosine phosphatase non-receptor type 22 (PTPN22) gene 1858 and 1123 sites single nucleotide polymorphism (SNP) with the susceptibility of primary immune thrombocytopenia (ITP).

**Method:** Database searched includes PubMed, Embase, Web of Science, CNKI, CBM, VIP and WanFang Data. The retrieval period is from the establishment of the database to 30 June 2021. After screening articles according to inclusion and exclusion criteria, the data were extracted and methodological quality of the included studies was evaluated. Meta-analysis was performed using RevMan 5.4 and Stata 16.0 software. The combined OR value and its 95%CI were calculated. Sensitivity analysis and publication bias assessment were performed. Trial sequential analysis (TSA) was performed using TSA 0.9.5.10 Beta software.

**Results:** A total of 10 studies with 10 articles were included, with a total of 932 cases and 2,112 controls. The results of meta-analysis showed that for SNP1858, the susceptibility of TT genotype to ITP was 5.01 times higher than CC genotype [95%CI (1.81, 13.86), *p* = 0.002]. For SNP1123, G allele carriers were more susceptible to ITP than C allele carriers [OR = 1.23, 95%CI (1.05, 1.45), *p* = 0.01], and GG genotype carriers were 1.51 times more susceptible to ITP than CC genotype carriers [95%CI (1.11, 2.06), *p* = 0.009]. Although the results are statistically significant, the results of sensitivity analysis showed certain limitations of stability, and the TSA analysis still indicated the possibility of false positive. No significant publication bias was observed.

**Conclusion:** PTPN22 gene SNP1858 (rs2476601) and SNP1123 (rs2488457) polymorphisms are associated with susceptibility to primary immune thrombocytopenia. Due to the limitation of the number and quality of the included studies, the above conclusions need to be verified by more high-quality studies.

## Introduction

Primary immune thrombocytopenia (ITP) is a common acquired hematologic autoimmune disease. It is one of the most common hemorrhagic disease which is caused by decrease in platelet count (platelet count <100×10^9^/L) (Kistangari and McCrae, 2013). ITP can affect individuals of all ages, which the incidence rate reaches the peak in children and the elder people (Monteagudo et al., 2019). In clinic, bleeding is the most common manifestation of ITP. Besides, evidence showed that the risk and associated morbidity of bleeding are both the highest in elder patients (Kistangari and McCrae, 2013; Monteagudo et al., 2019). The pathogenesis of ITP is complex and has been demonstrated associate with changes in humoral and cellular immunity (Lee and Lee, 2016). Study has shown that ITP is caused by the reaction between the antibodies and the glycoproteins (glycoproteins IIb/IIIa, Ib/IX, etc.) which is expressed on platelets and megakaryocytes, resulting in shortening survival of circulating platelets and affecting platelet production (Toltl et al., 2011). Moreover, the reduction in the function and number of regulatory T cells and the role of cytotoxic T cells may contribute to the pathogenesis of ITP, some important genes were also included in the production of platelets, dysfunction of those genes will contribute to ITP (Semple et al., 2010).

The etiology and pathogenesis of ITP also involve a variety of genetic risk factors. Studies has shown that PTPN22 gene has a significant role in the regulation of signal transduction through T-cell receptors (TCR) (Hesham et al., 2020) which is highly associated with the pathogenesis of ITP. Expression of PTPN22 mutation is increased in patients with ITP (D'Silva et al., 2010). Moreover, some studies had also shown that PTPN22 SNP1858 C > T and PTPN22 SNP1123 C > G have important role in human with ITP, which also shows that polymorphism in the PTPN22 gene may play a key role in genetic susceptibility and disease progression of ITP in children (Anis et al., 2011).

PTPN22 gene has been proved by researchers to play an important role in type Ⅰ diabetes (Bottini et al., 2006) and rheumatoid arthritis (Carmona and Martín, 2018). However, there is no relevant research to analyze the relationship between PTPN22 and ITP. Moreover, there are many case-control studies of PTPN22 SNP1858 C > T and PTPN22 SNP1123 C > G susceptibility to primary immune thrombocytopenia in these years. Due to the uneven quality of similar studies, or small sample size, or regional and ethnic differences, their conclusions are different, which limits the credibility of the results. The purpose of this study was to conduct a meta-analysis of case-control studies on the correlation between SNP1858, SNP1123 polymorphisms of PTPN22 gene and susceptibility to ITP, in order to provide more reliable evidences for basic research and clinical treatment.

## Methods

### Inclusion Criteria

(1) Case-control studies which evaluated the association between SNP1858 and SNP1123 polymorphisms of PTPN22 gene and susceptibility to ITP.
(2) The case group of the case study consisted of patients with primary immune thrombocytopenia diagnosed clinically and pathologically, and the control group consisted of healthy people with no restrictions on gender and race.
(3) Frequencies of alleles or genotypes in control groups and case groups could be obtained from the included articles.

### Exclusion Criteria

(1) Analysis data is incomplete or missing, and contact with the original author is not available.
(2) The subject of the original study is not human.
(3) Repeated publication and retrieved article.
(4) For repeated publications by the same author, the one with the highest quality and the largest sample size was selected.

### Search Strategy

PubMed, Embase, Web of Science, CNKI, CBM, VIP, and WanFang Data databases were searched. The retrieval period is from the establishment of the database to 30 June 2021. The references of the included literatures were retrospectively reviewed to supplement the relevant articles. The retrieval is carried out by the combination of free words and subject words. Search terms include ITP, Idiopathic Thrombocytopenic Purpura, Immune Thrombocytopenia, PTPN22, Protein tyrosine phosphatase non-receptor type 22, PTPN22 polymorphism, PTPN22 variants, PTPN22 genotype. Taking PubMed as an example, its specific retrieval strategies are shown in Table 1.

**TABLE 1:** Article search strategy (Taking PubMed as an example).

#1	“Purpura, thrombocytopenic, Idiopathic" [Mesh]
#2	ITP
#3	Idiopathic Thrombocytopenic Purpura
#4	Immune Thrombocytopenia
#5	Autoimmune Thrombocytopenia Purpura
#6	#1 OR #2 OR #3 OR #4 OR #5
#7	PTPN22
#8	Protein tyrosine phosphatase non-receptor type 22
#9	#7 OR #8
#10	#6 AND #9

### Article Screening and Data Extraction

Two researchers independently screened the articles, extracted the data and cross-checked them. Disputes, if any, shall be settled through discussion or negotiation with a third party. When screening the articles, the title was first read, and after excluding obviously irrelevant literature, the abstract and the full text were further read to determine whether to be included. If necessary, contact the original study author by email or telephone for undetermined but important information for this study.

### Literature Quality Evaluation

The quality of the included case-control study was evaluated by referring to The Newcastle-Ottawa Scale (NOS), including whether the definition and diagnosis of the case are appropriate, whether the case is representative, whether the selection and definition of the control are clear, whether the case is comparable with the control, whether the investigation and evaluation methods of exposure are the same, whether the investigation methods of case and control are the same, and whether there is no response rate (GA Wells et al., 2021).

### Statistical Method

This meta-analysis was performed using RevMan 5.4 and Stata 16.0 software with the count data conveyed in the form of odds ratio (OR) and its 95% confidence interval (95%CI) of alleles and genotypes. The calculation of heterogeneity between the results originated from each selected study was done utilizing chi-square test (test level: *α* = 0.10). Encountering small statistical homogeneity between studies (*p* > 0.10), a fixed-effect model was used in the meta-analysis. Contrariwise, when huge statistical homogeneity between studies were exposed (*p* < 0.10), a random-effect model was used for meta-analysis. We also had Hardy-weinberg balance calculated based on the data of controls, and *p* < 0.05 was considered statistically significant. We performed sensitivity analysis with one-by-one exclusion method to see if one study greatly influenced the overall results. The publication bias was evaluated using Begg’s test and Egger’s test. *p* > 0.05 was considered the publication bias was minute. TSA was used to analysis the problem of random errors such as false positives and false negatives in the repeated updates of meta-analysis, and to calculate the sample size needed to draw a definite conclusion by TSA 0.9.5.10 Beta software (Wang et al., 2013).

## Results

### Article Retrieval Results and Quality Evaluation

**FIGURE 1:**
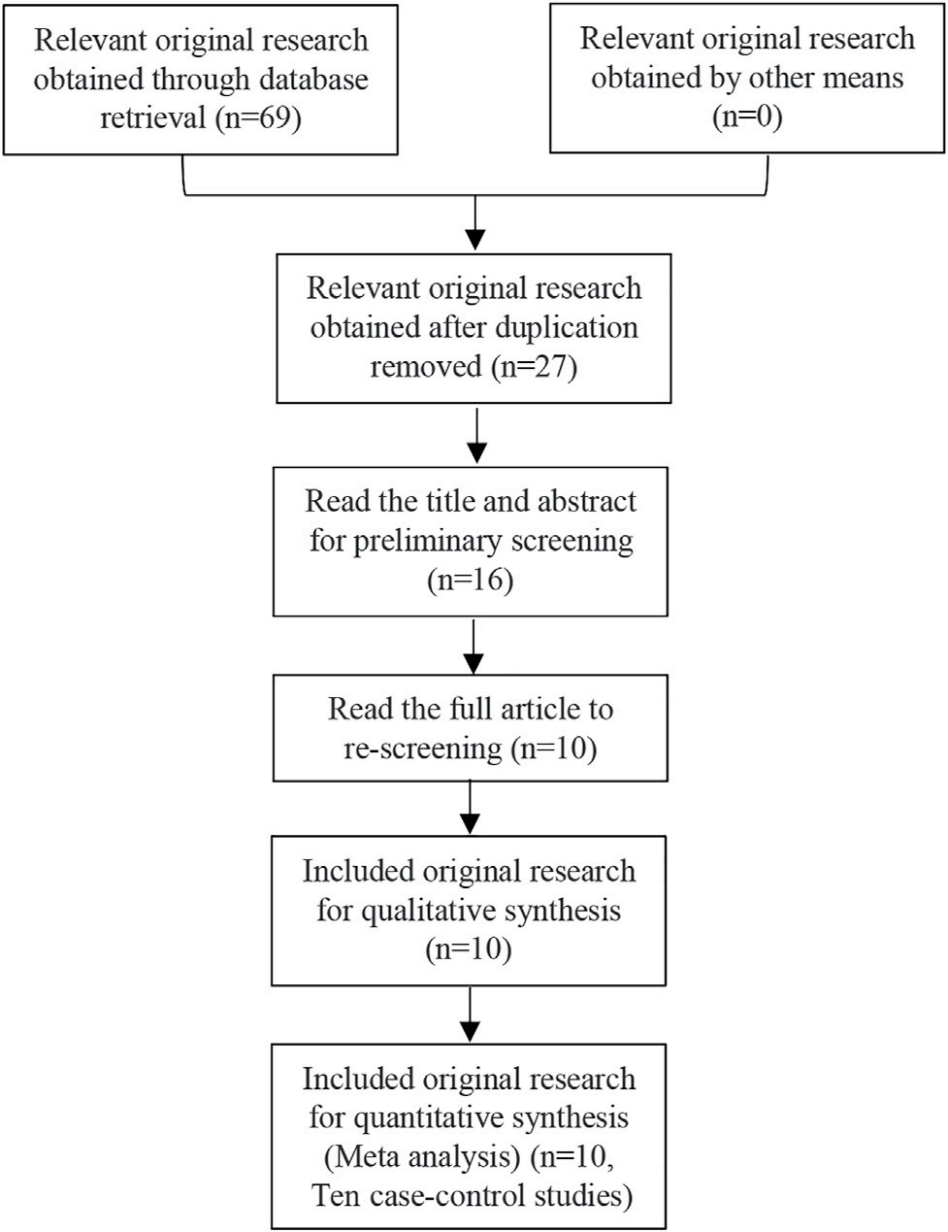
Article screening process and results.

69 related articles were initially detected, and 10 articles (D'Silva et al., 2010; Anis et al., 2011; Elhoseiny et al., 2013; Ge et al., 2013; He, 2014; Sun et al., 2015; Lioger et al., 2016; Li et al., 2017; Hesham et al., 2020; Wan et al., 2020) including 10 case-control studies, were finally included after layer by screening step by step. There were 932 cases and 2,112 control cases included. Among them, there were five articles involving PTPN22 gene SNP1858, including 323 cases and 1,316 controls. There were five articles involving PTPN22 gene SNP1123, with a total of 609 cases and 796 controls. As for ITP patients, there were 6 studies involved children in this meta-analysis. Considering the five studies related to SNP1858, there were 215 children and 108 adults that be involved in. Considering the five studies related to SNP1123, there were 188 children and 421 adults that involved in. The aeticle screening process and results are shown in Figure 1, and the basic characteristics of the included studies are shown in Table 2 and Table 3. The results of literature quality evaluation showed that the included studies had clear diagnostic criteria, comparable cases and controls, reasonable genetic testing methods, and clear results. Among all the included articles, five articles were about East Asians, three articles were about Africans, 1 article was about North Americans and 1 article was about Europeans. Allele data were available in all 10 studies, and the control groups in four studies met Hardy-Weinberg equilibrium, while the control groups in six studies did not meet Hardy-Weinberg equilibrium.

**TABLE 2:** Basic features of the included articles about research of correlation between SNP1858 and ITP susceptibility.

Included Studies	Country	Race	Test Methods	Number of People (Case Group / Control Group)	Genotype of Case Group			Genotype of Control Group			Hardy–Weinberg Equilibrium
					TT	TC	CC	TT	TC	CC	
KJ D'Silva et al. (2010)	The United States	American	PCR-RFLP	45/926	2	10	33	6	149	771	No
SK Anis et al. (2011)	Egypt	African	PCR-RFLP	50/50	3	10	37	0	3	47	No
Elhoseiny SM et al. (2013)	Egypt	African	PCR-RFLP	60/100	4	12	44	0	8	92	No
Lioger B et al. (2016)	French	European	Unmentioned	88/160	0	15	73	2	28	130	No
Hesham M et al. (2020)	Egypt	African	PCR-RFLP	80/80	10	44	26	0	72	8	Yes

**TABLE 3:** Basic features of the included articles about research of correlation between SNP1123 and ITP susceptibility.

Included Studies	Country	Race	Test Methods	Number of People (Case Group / Control Group)	Genotype of Case Group			Genotype of Control Group			Hardy–Weinberg Equilibrium
					GG	GC	CC	GG	GC	CC	
Ge J et al. (2013)	China	East Asian	PCR-MALDI-TOF-MS	191/216	29	81	81	20	87	109	No
He L (2014)	China	East Asian	PCR-RFLP and PCR-SSP	100/100	32	54	14	33	56	11	Yes
Sun Z et al. (2015)	China	East Asian	PCR-RFLP and PCR-SSP	98/100	27	65	6	32	58	10	Yes
Li F et al. (2017)	China	East Asian	PCR-MALDI-TOF-MS	120/80	50	20	50	34	6	40	Yes
Wan H et al. (2020)	China	East Asian	PCR-MALDI-TOF-MS	100/300	26	10	64	42	43	215	No

### Study on the Susceptibility Between SNP1858 and ITP

#### The Relationship Between the Genotype TT *vs* CC and ITP

**FIGURE 2:**
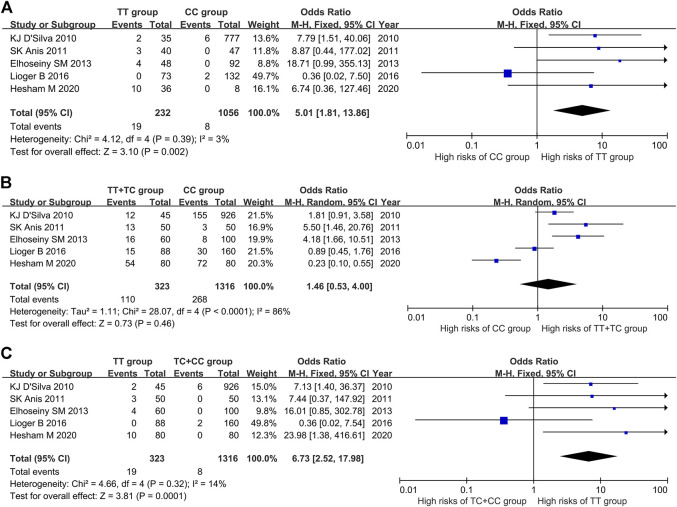
Meta-analysis of correlation between PTPN22 gene SNP1858 and ITP. **(A)** TT vs CC. **(B)** TT + TC vs CC. **(C)** TT vs TC + CC.

The results of heterogeneity test showed that there was no statistical heterogeneity among the research results (χ^2^ = 4.12, *p* = 0.39), so the fixed effect model was used for combined analysis. Taking genotype TT as the exposure factor and genotype CC as the non-exposure factor, the meta-analysis results showed that the incidence risk of ITP in the population with genotype TT was higher than that in the population with genotype CC [OR = 5.10, 95%CI (1.81, 13.86), *p =* 0.002], and the results were statistically significant (Figure 2A).

#### The Relationship Between the Genotype TT + TC *vs* CC and ITP

The results of heterogeneity test showed that there was statistical heterogeneity among the research results (χ^2^ = 28.07, *p* < 0.0001), so the random effect model was used for combined analysis. The results of meta-analysis showed that there was no significant difference in the risk of ITP in the dominant model TT + TC *vs* CC group [OR = 1.46, 95%CI (0.53, 4.00), *p* = 0.46] (Figure 2B).

#### The Relationship Between Genotype TT *vs* TC + CC and ITP

The results of heterogeneity test showed that there was no statistical heterogeneity among the research results (χ^2^ = 4.66, *p* = 0.32), so the fixed effect model was used for combined analysis. The results of meta-analysis showed that there was significant difference in the incidence risk of ITP among the population of the recessive model TT *vs* TC + CC group. The incidence risk of ITP in the population of TT genotype was higher than that in the population of TC + CC genotype [OR = 6.73, 95%CI (2.52, 17.98), *p* = 0.0001] (Figure 2C).

#### The Relationship Between SNP1858 Polymorphism of PTPN22 Gene and Susceptibility to ITP

The results of meta-analysis showed that the susceptibility to ITP of patients with T allele was higher than that of patients with C allele [OR = 1.86, 95%CI (0.90, 3.84), *p* = 0.10], the susceptibility to ITP of patients with TT genotype was 5.01 times higher than that of patients with CC genotype [95%CI (1.81, 13.86), *p* = 0.002], and the susceptibility to ITP of patients with TT genotype was 5.39 times higher than that of patients with TC genotype [95%CI (2.01, 14.47), *p* = 0.0008] (Table 4).

**TABLE 4:** The relationship between SNP1858 and SNP1123 polymorphism of PTPN22 gene and ITP susceptibility.

Genotype	SNP1858			SNP1123		
	OR	95%CI	*P*	OR	95%CI	*P*
A *vs* B	1.86	(0.90, 3.84)	0.10	1.23	(1.05, 1.45)	0.01
AA *vs* BB	5.01	(1.81, 13.86)	0.002	1.51	(1.11, 2.06)	0.009
AA *vs* AB	5.39	(2.01, 14.47)	0.0008	1.09	(0.66, 1.82)	0.73
AB *vs* BB	1.23	(0.46, 3.30)	0.67	1.21	(0.90, 1.63)	0.21
(AA + AB) *vs* BB	1.46	(0.53, 4.00)	0.46	1.34	(1.05, 1.72)	0.02
AA *vs* (AB + BB)	6.73	(2.52, 17.98)	0.0001	1.24	(0.85, 1.81)	0.27

For SNP1858, A represents T, B represents C. For SNP1123, A represents G, B represents C.

### The Research on the Susceptibility Between SNP1123 and ITP

#### The Relationship Between the Genotype GG *vs* CC and ITP

**FIGURE 3:**
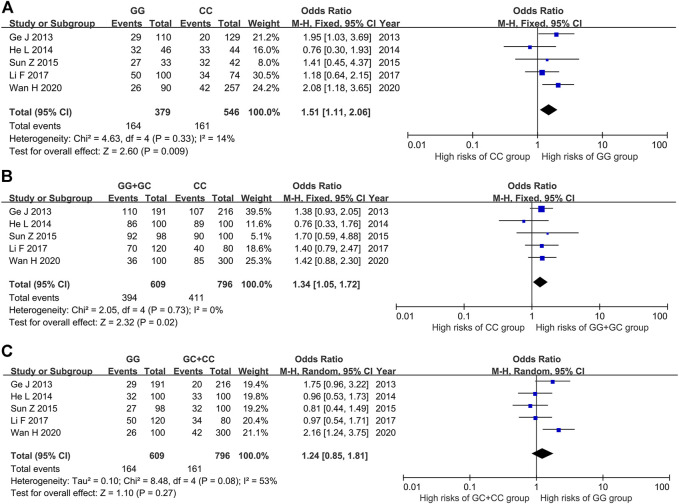
Meta-analysis of correlation between PTPN22 gene SNP1123 and ITP. **(A)** GG vs CC. **(B)** GG + GC vs CC. **(C)** GG vs GC + CC.

The results of heterogeneity test showed that there was no statistical heterogeneity among the research results (χ^2^ = 4.63, *p* = 0.33), so the fixed effect model was used for combined analysis. Taking genotype GG as the exposure factor and genotype CC as the non-exposure factor, the meta-analysis results showed that the risk of ITP in the population with genotype GG was higher than that in the population with genotype CC [OR = 1.51, 95%CI (1.11, 2.06), *p* = 0.009], and the results were statistically significant (Figure 3A).

#### The Relationship Between the Genotype GG + GC *vs* CC and ITP

The results of heterogeneity test showed that there was no statistical heterogeneity among the research results (χ^2^ = 2.05, *p* = 0.73), so the fixed effect model was used for combined analysis. The results of meta-analysis showed that there was a statistically significant difference in the risk of ITP in the dominant model GG + GC *vs* CC group. The risk of ITP in the GG + GC genotype was higher than that in the CC genotype [OR = 1.34, 95%CI (1.05, 1.72), *p* = 0.02] (Figure 3B).

#### The Relationship Between the Genotype GG *vs* GC + CC and ITP

The results of heterogeneity test showed that there was statistical heterogeneity among the research results (χ^2^ = 8.48, *p* = 0.08), so the random effect model was used for combined analysis. The results of meta-analysis showed that there was no significant difference in the risk of ITP between the implicit model GG *vs* GC + CC group [OR = 1.24, 95%CI (0.85, 1.81), *p* = 0.27] (Figure 3C).

#### The Relationship Between SNP1123 Polymorphism of PTPN22 Gene and Susceptibility to ITP

The results of meta-analysis showed that the susceptibility to ITP of patients with G allele was higher than that of patients with C allele [OR = 1.23, 95%CI (1.05, 1.45), *p* = 0.01]. The susceptibility to ITP of patients with GG genotype was 1.51 times higher than that of patients with CC genotype [95%CI (1.11, 2.06), *p =* 0.009] (Table 4).

### Sensitivity Analysis

Sensitivity analysis was performed by one-by-one exclusion method. Taking TT *vs* CC genotype of SNP1858 as an example, the maximum combined OR value was 9.61 [95%CI (2.53, 36.49)] (excluding Lioger B 2016) and the minimum value was 3.69 [95%CI (1.20, 13.34)] (excluding Elhoseiny SM et al., 2013). Taking GG *vs* CC genotype of SNP1123 as an example, the maximum combined OR value was 1.65 [95%CI (1.19, 2.30)] (excluding He L 2014) and the minimum value was 1.33 [95%CI (0.92, 1.93)] (excluding Wan H 2020). Sensitivity analysis showed that the meta-analysis results were relatively stable.

### Publication Bias Analysis

Publication bias analysis of PTPN22 gene SNP1858 and SNP1123 polymorphism and ITP susceptibility using Egger’s test was shown as Table 5. All the *p* values were higher than 0.05, indicating no publication bias was observed for the results relating ITP risk to PTPN22 gene polymorphism in the included studies. Taking TT *vs* CC group of SNP1858 as an example, Begg’s test showed that *Z* = 0.24, *p* = 0.806 > 0.05, which also indicated no publication bias.

**TABLE 5:** Publication bias analysis of PTPN22 gene SNP1858 and SNP1123 polymorphism and ITP susceptibility.

Genotype	SNP1858			SNP1123		
	Std. Err	*t*	*P*	Std. Err	*t*	*P*
A *vs* B	0.16	2.37	0.098	0.95	1.94	0.148
AA *vs* BB	0.03	1.77	0.175	0.45	1.36	0.268
(AA + AB) *vs* BB	0.48	2.03	0.136	0.23	3.09	0.054
AA *vs* (AB + BB)	0.04	1.48	0.236	0.51	0.20	0.853

For SNP1858, A represents T, B represents C. For SNP1123, A represents G, B represents C.

### Trial Sequential Analysis

**FIGURE 4:**
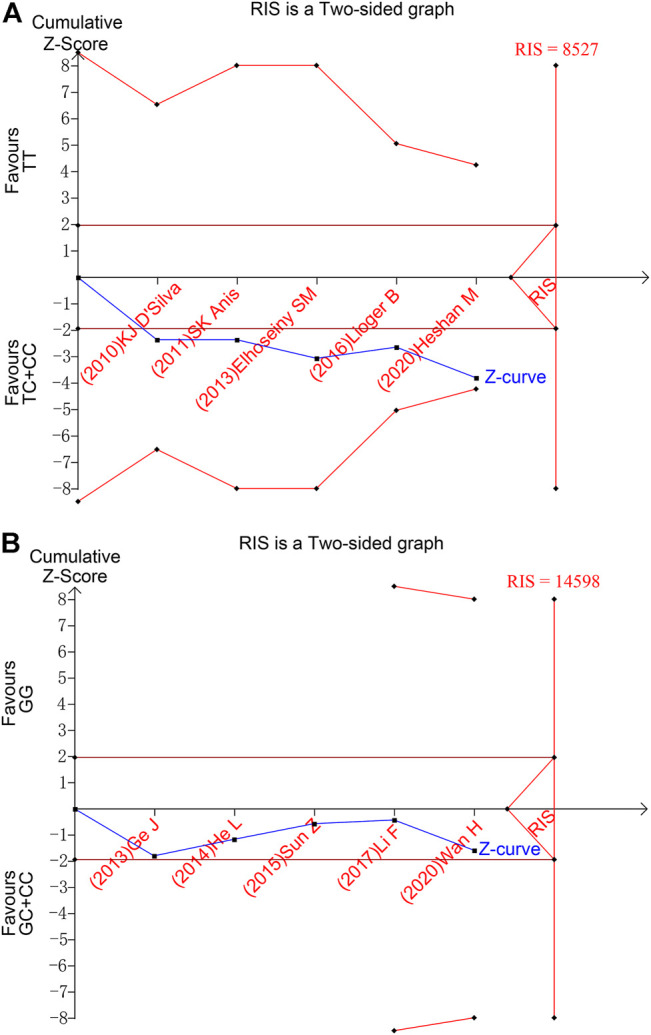
Trial sequential analysis results of TT vs TC + CC of SNP1858 group and GG vs GC + CC of SNP1123 group. **(A)** TT vs TC + CC of SNP1858. **(B)** GG vs GC + CC of SNP1123.

To evaluate the accuracy of meta-analysis, trial sequential analysis was performed. Taking SNP1858 TT *vs* TC + CC group as an example, the accumulated information amount did not reach the Required Information Size (RIS), and the Z-curve was about to intersect the TSA boundary line but have not yet (Figure 4A). The result of SNP1123 GG *vs* GC + CC group also showed the same tendency (Figure 4B). The figure demonstrated that the result of meta-analysis showed positive results, but the Z curve did not either cross the TSA boundary value or reach the RIS, indicating that the results still have the possibility of false positive. Therefore, more follow-up case-control studies are still needed to further verify this result.

## Discussion

In this study, PTPN22 gene polymorphism and ITP susceptibility were systematically evaluated. The results showed that the OR value of ITP susceptibility of TT genotype relative to CC genotype in the SNP1858 polymorphism of PTPN22 gene was 5.01, with 95%CI (1.81, 13.86) and *p* = 0.002, indicating the risk of ITP in the population with TT genotype was higher than that in the population with CC genotype. At the same time, in the SNP1123 polymorphism of PTPN22 gene, the OR value of ITP susceptibility of G allele relative to C allele was 1.23, 95%CI (1.05, 1.45), *p* = 0.01. The risk of ITP in population with homozygous GG genotype was 1.51 times higher than that in CC genotype [95%CI (1.11, 2.06), *p* = 0.009], indicating that the G allele at SNP1123 of PTPN22 may increase the susceptibility to ITP.

Nearly 100 genes are associated with autoimmune diseases. PTPN22 has been confirmed to be associated with a variety of autoimmune diseases, including type Ⅰ diabetes, rheumatoid arthritis and systemic lupus erythematosus (Hinks et al., 2005). The most likely pathway is related to activation and effector function of PTPN22 on T cells, so as to regulate and affect human immune diseases. PTPN22 is a cytoplasmic non receptor protein tyrosine phosphatase, which is mainly expressed in cells of hematopoietic origin (Cloutier and Veillette, 1996). The initiation of Src tyrosine kinases Lck and Fyn can phosphorylate immunoreceptor tyrosine based activation motifs (ITAMs) in the zeta chain of CD3 associated with TCR. ITAM phosphorylation can recruit and phosphorylate TCR. After antigenic peptide being presented to TCR, Src tyrosine kinases, Lck and Fyn participate in the phosphorylation of immunoreceptor tyrosine-based activatory motifs (ITAMs) identified in CD3 and zeta chains that are associate with TCR. Subsequently, zeta-associated protein kinase of 70 kDa (ZAP70) is recruited and phosphorylated, which encourages downstream signaling that results in gene expression and effector functions (Salmond et al., 2009; Brownlie and Zamoyska, 2013). PTPN22, as a cytoplasmic non-receptor protein tyrosine phosphatase (Cloutier and Veillette, 1996), mediates TCR proximal signaling by dephosphorylating Lck Y394 (Chiang and Sefton, 2001; Wiede et al., 2011), which is presented in the key tyrosine residues within the kinase domain (Salmond et al., 2009). It is said that the phosphorylation of Lck Y394 is responsible for optimizing Lck kinase activity (Brownlie et al., 2018). Therefore, PTPN22 restricts the TCR proximal signaling. Moreover, it has been witnessed that PTPN22 can also influence both “inside-out” and “outside-in” signaling which T cells partake in (Salmond et al., 2014; Burn et al., 2016). When PTPN22 mutates, the activity and function of Src family kinases will be changed by the kinase domain (such as Lck394) and C-terminal (Salmond et al., 2009). When CSK cannot be phosphorylated, Lck505 residue and Src homology (SH) two domain will lose inhibition, so that intermolecular connection cannot be formed and Lck cannot maintain a closed conformation. Therefore, immune receptor tyrosine activating elements (ITAMs) cannot form normally, which affects the normal operation of the immune system (Brownlie and Zamoyska, 2013).

PTPN22 is also considered to be related to Csk composition in B cell. However, the intrinsic role of PTPN22 in B cell ontogeny, activation and dependent immune response has not been effectively confirmed. The study showed that there was no difference between the total number of peripheral blood B cells in PTPN22 knockout mice and that in normal mice (Hasegawa et al., 2004). Similarly, Zikherman et al. found that the lack of PTPN22 had no effect on the number of follicular mature cells, T1, T2, cell number in marginal zone B cell chamber and plasma cells (Zikherman et al., 2009). However, high expression of PTPN22 was found in chronic lymphoblastic leukemia (CLL) mother cells, suggesting that PTPN22 may play a role in malignant B cell signal transduction. The knockout of PTPN22 or the inhibition of its enzymatic function can promote BCR triggered CLL cell apoptosis, indicating that PTPN22 may protect malignant cells from antigen receptor induced death by inhibiting or promoting different pathways downstream of BCR (Negro et al., 2012).

As for the two SNPs in PTPN22 involved in our study, some experimental studies have proved that their mechanism of action may be related to the pathogenesis of ITP. Previous studies have shown that SNPs generated in the binding region of transcription factors may affect the binding characteristics of transcription factors, leading to differences in binding strength of transcription factors on alleles, which could cause differences in susceptibility of some diseases among populations (Brodie et al., 2016). PTPN22 is one of the major molecules involved in T cell regulation. Studies had shown that the function of SNP rs2476601 is a negative regulator of T cell activation (Begovich et al., 2004). Therefore, up-regulation and down-regulation of this SNP will result in a difference in the number of activated T cells (Schulz et al., 2020), which may lead to the changes in the immune environment in the human body and lead to differences in susceptibility to ITP. SNP rs2488457 may have the similar mechanism, but more research is needed to prove this viewpoint in the future.

In this study, no significant publication bias was found through Begg’s test funnel plot and Egger’s test analysis. Therefore, the results have a certain credibility. However, the following limitations may still exist. First, the studies included in this article are all published studies, and the languages are limited to Chinese and English. Potential publication and language bias may still affect the results of meta-analysis. Secondly, the research on SNP1858 and SNP1123 polymorphisms of PTPN22 gene and disease susceptibility mainly focuses on solid tumors at present, and the number of studies on blood system is small, resulting in a limited number of studies and a limited number of cases and controls included in this study, thus reducing the representativeness of the conclusion, which is also indicated by TSA results. At the same time, due to the limitation of the number of studies included in this article, subgroup analysis on race and genotyping methods cannot be carried out. Besides, many of the studies we included have both children and adults involved in a single study, and did not provide data of different age groups separately. As a consequence, the total genotype data of different age groups is limited, and subgroup analysis based on age could not be carried out, which reduces the comprehensiveness of the results.

In conclusion, as a susceptibility gene of autoimmune diseases, PTPN22 gene plays an important role in ITP. SNP1858 and SNP1123 polymorphisms of PTPN22 gene are related to ITP susceptibility. For SNP1858, population with TT genotype has high risk of ITP than those with CC genotype. For SNP1123, allele G may be a risk factor for ITP susceptibility. As an autoimmune disease whose pathogenesis is not completely clear, studying the correlation between ITP pathogenesis and PTPN22 gene SNP1858 and SNP1123, and then understanding the pathogenesis of ITP at the gene level can provide new ideas enlightening research about ITP mechanism and treatment. In the future, it is expected to carry out more case-control studies with multi-centers, large samples and good homogeneity, in order to more scientifically and accurately evaluate the correlation between PTPN22 gene polymorphism and ITP susceptibility, and provide more reliable evidence for basic research and clinical treatment.

## Data Availability

The original contributions presented in the study are included in the article/Supplementary Material, further inquiries can be directed to the corresponding author.
